# Future oriented group training for suicidal patients: a randomized clinical trial

**DOI:** 10.1186/1471-244X-9-65

**Published:** 2009-10-07

**Authors:** Wessel van Beek, Ad Kerkhof, Aartjan Beekman

**Affiliations:** 1 Symfora groep, Hilversum; EMGO Institute for Health and Care Research, Amsterdam, The Netherlands; 2 Vrije Universiteit, Dpt Clinical Psychology, Amsterdam; EMGO Institute for Health and Care Research, Amsterdam, The Netherlands; 3 Vrije Universiteit, Dpt Psychiatry, Amsterdam; EMGO Institute for Health and Care Research, Amsterdam, The Netherlands; 4 Symfora groep, locatie Rembrandthof, Postbus 219, 1200 AE Hilversum, The Netherlands

## Abstract

**Background:**

In routine psychiatric treatment most clinicians inquire about indicators of suicide risk, but once the risk is assessed not many clinicians systematically focus on suicidal thoughts. This may reflect a commonly held opinion that once the depressive or anxious symptoms are effectively treated the suicidal symptoms will wane. Consequently, many clients with suicidal thoughts do not receive systematic treatment of their suicidal thinking. There are many indications that specific attention to suicidal thinking is necessary to effectively decrease the intensity and recurrence of suicidal thinking. We therefore developed a group training for patients with suicidal thoughts that is easy to apply in clinical settings as an addition to regular treatment and that explicitly focuses on suicidal thinking. We hypothesize that such an additional training will decrease the frequency and intensity of suicidal thinking.

We based the training on cognitive behavioural approaches of hopelessness, worrying, and future perspectives, given the theories of Beck, McLeod and others, concerning the lack of positive expectations characteristic for many suicidal patients. In collaboration with each participant in the training individual positive future possibilities and goals were challenged.

**Methods/Design:**

We evaluate the effects of our program on suicide ideation (primary outcome measure). The study is conducted in a regular treatment setting with regular inpatients and outpatients representative for Dutch psychiatric treatment settings. The design is a RCT with two arms: TAU (Treatment as Usual) versus TAU plus the training. Follow up measurements are taken 12 months after the first assessment.

**Discussion:**

There is a need for research on the effectiveness of interventions in suicidology, especially RCT's. In our treatment program we combine aspects and interventions that have been proven to be useful in the treatment of suicidal thinking and behavior.

**Trial registration:**

ISRCTN56421759

## Background

Suicide has a low prevalence in the general population, but suicide ideation is remarkably common [1]. In The Netherlands about 10% of the general public reported that they ever had suicidal thoughts [2], and Casey et al. [3] found no differences amongst several European countries. When these patients enter treatment, they are confronted with a commonly held misconception amongst health care workers that suicidal thinking and behavior will vanish when underlying psychiatric problems are treated. We have very good reasons to believe this is not the case. Suicidal thinking fluctuates over time [4,5], and reoccurs in the majority of depressed individuals in a consecutive episode [6]. In a study amongst formerly suicidal patients, Williams et al. [7] showed that problem-solving abilities and autobiographical memory specificity, commonly associated with suicidal thinking and behavior, deteriorate when the patient's mood lowers again. Suicidality appears to become a syndrome irrespective of underlying psychiatric morbidity [8]. This is a reason for Oquendo et al. [9] to plea for a separate diagnostic category in the forthcoming DSM classification manual. A distinct psychiatric problem, which needs a specific intervention.

Clinicians in psychiatry are increasingly aware of the need for systematically assessing suicidal risk, but they lack the tools for addressing suicidal thinking as a specific goal in treatment. There is a shortage of well described, evidence based treatment methods for suicidal behavior and suicide ideation. A few randomized clinical trials focussing on self harm and suicidal behaviour have been published, like MACT [10] (Manual Assisted Cognitive- Behavior Therapy) and the study on cognitive therapy for suicide attempters by Brown et al. [11]. There are studies on suicidality as a component of treatment programs for borderline patients, like Dialectical Behavioral Therapy [12,13], Schema Focussed Therapy [14], and Mentalization Based Treatment [15]. Most of these interventions have been applied in the Netherlands, but they all are developed for specialized settings and specific patient groups. A reason for us to develop an intervention for a broad group of patients with suicidal thoughts. This new intervention should be easy to implement as an add on intervention. Therefore, it should not require highly specialized therapists.

The most consistent and convincing theories on suicidal thinking and behavior include hopelessness, so this should be the core component of our intervention. According to Beck [16] three variables constitute the so called negative triad: hopelessness, self-esteem and a negative perception of the environment. Hopelessness is considered to be the best predictor or indicator of the risk of suicidal behaviour [17]. Research shows that especially lack of positive future expectancies, as a part of hopelessness, is an important factor in developing suicidal ideations and behavior [18]. MacLeod et al. [19] have shown that specifically a deficit of positive anticipation about the future relates to hopelessness and discriminates between parasuicidal and non-parasuicidal groups. Parasuicidal patients show an absence of anticipation of pleasurable future events, but not an increased anticipation of unpleasant events [20]. Lack of positivity seems to be related to cluster B disorders, especially borderline and dissocial personality disorder [21]. MacLeod hypothesized that this shortage of positivity might reflect a lack of available sources or rewarding and enjoyable experiences, a cognitive inaccessibility of representations of future positive outcomes or it may represent an inability to derive pleasure from what are normally enjoyable events [22]. Research among older individuals by Hirsch et al. [23] reveals that positive future orientation is associated with less current and less worst point suicide ideation. These authors regret that no cognitive based treatment has focused specifically on enhancing future orientation.

Another element of any new intervention for suicidal individuals should be problem solving. According to Hawton et al. [24] forms of problem solving therapy are promising in the treatment of suicidal patients. Recent research by Eskin et al. [25] showed significant decrease of suicide risk when adolescents and young adults received PST. Consistent evidence has shown that people who attempt suicide have poor problem solving skills [26,27] and problem solving therapy showed to reduce levels of depression and hopelessness in patients who have attempted suicide [28]. A study among suicide attempters by Jollant et al. [29] shows that decision making is impaired in this group, evaluated in a period in which the participants had no axis I disorder. Several attempts have been made to influence problem-solving skills, like MACT [30], STEPPS [31] (Systems Training for Emotional Predictability and Problem Solving, and BATD [32] (Behavioral Activation Treatment for Depression). In general health practice Problem Solving Therapy (PST), developed by Nezu, Nezu and Perri [33], has proven to be helpful and it is one of the treatment methods in the Dutch Multidisciplinary Treatment Guidelines for Depression [34].

Some other available interventions have a stronger focus on dysfunctional cognitions, like the time-limited approach by Rudd, Joiner & Rehad [35], and cognitive therapy for suicide attempters, evaluated in a RCT by Brown et al. [36]. These authors developed a 10 week program in which they combined basic cognitive therapy with elements like safety seeking and behavioral experiments. They found a 50% lower reattempt rate in their cognitive therapy sample, even after 18 months.

Suicidal behavior is characterized by isolation and social detachment [37]. As a result local and governmental incentives to encourage health-seeking behavior and to increase social support were developed. Examples are the Scottish 'Choose Life' program, and Australia's 'Social Inclusion Suicide Prevention Initiative'. On a smaller scale we encourage the participants in our project to seek for a coach or buddy to support them during the training. We stimulate patients to involve partners or friends. We are working on a pool of volunteers that can be contacted when participants have no one who can act as their personal coach. This supportive role is an element in other programs as well, like in the Community Reinforcement Approach [38].

This led to the cornerstones that we used to develop an intervention which we called Future Oriented Group Training. The training addresses hopelessness and lack of future thinking, and includes elements from cognitive therapy and problem solving therapy. Furthermore, a main goal is to break through the social isolation most participants got stuck in. In this article we describe the outlines of the training and our research project.

## Methods/Design

### Design

In order to evaluate the effectiveness of our intervention program we carry out a pragmatic randomized clinical trial (RCT). The participants are randomly assigned to either treatment as usual (TAU), or treatment as usual plus our additional treatment (TAU+). There are three assessments: when participants enter the project (pre-measurement), after three month (post measurement), and the follow up measurement carried out one year after the baseline measurement.

This research has been approved by the METiGG, the medical-ethical committee for research in mental health care settings in the Netherlands.

### Participants

People enter this project in several ways. The main stream of participants (aged 18-65 years) enters the project after an initial assessment in two psychiatric hospitals in The Netherlands, both in-patients and outpatients. A smaller sample is recruited from the existing pool of patients already in treatment and who were referred to the program due to suicide ideation.

The intervention is open for patients with suicidal ideation, irrespective of comorbid psychiatric disorders. Patients in an acute manic or psychotic state and those who seek treatment primarily because of drugs problems are excluded. Suicidal behavior is not a reason to exclude patients. Participants are required to speak and read Dutch sufficiently to take part in the study. All participants signed an informed consent form.

### Randomization

The randomization is conducted by an independent statistician. The researchers receive the outcome of the randomization by email and schedule the participants accordingly.

### Sample Size

The effect size deemed worthwhile to be detected by the study is *d *= 0.5. This is what is generally judged to be a clinically relevant effect size [39]. Power calculations are based upon a type I error α = 0.05, a power of 0.80, and an effect size of 0.5, imply a minimum of 63 participants in the groups. We calculated power to be sufficient for both intention-to-treat and completers analysis. Expecting that 80% of the patients in the 'suicide ideations group' are willing to participate (before randomization), and a drop out of 20% after randomization, we need to include 75 patients in each of the two groups to maintain 63 completers.

### Blinding

Given the nature of the intervention, it was impossible to blind the patients and the trainers as to which condition they participated in. The outcomes will be assessed by blinded interviewers.

### Experimental Condition

The patients with suicidal ideation who are randomly assigned to the TAU+ condition receive an additional intervention called Future Oriented Group Training. There are 4 to 8 participants in each group, and the sessions are led by one trainer. This intervention consists of three major elements: the training sessions, the workbook with a accompanying audio cd, and a website.

The main goal of this training is to decrease suicidal thinking by stimulating realistic future thinking and reducing hopelessness. The training promotes goal directed and future oriented behavior by combining cognitive therapy, problem solving therapy, and future thinking. This means that participants and trainers almost exclusively address things to come.

The 10 weekly group training sessions last one and a half hours each. They are organized as workshops. Participants listen to the trainer who explains and discusses relevant topics. The trainer asks for personal experiences, but remains on a practical and educational level. The trainer discusses general tendencies, and individual experiences are generalized and reformulated in terms of future oriented cognitions and behavior. How would this kind of thinking, or that way of behaving, influence one's chance of reaching future positive goals? And what can be done about this?

The exercises and texts included in the workbook promote realistic thinking and help participants to create a personal meaningful future, by accomplishing goals that make life worthwhile again. In the workbook notorious cognitive patterns among suicidal patients are challenged, like dichotomous thinking and external locus of control. The participants receive information about suicidal vulnerability and factors influencing this vulnerability, for instance perfectionism, social isolation, and alcohol and drug abuse. The workbook discusses several practical steps, like making a survival plan, and creating a scrapbook with positive elements from their present and their past, in order to find strength when they feel hopeless. The workbook comes with an audio cd, with additional exercises that are in line with the contents of the workbook.

The supplementary website provides information about the training and the research project. It gives directions about the practical steps participants can take, like where to find help for their alcohol problems. It also provides means to discuss the training and exchange information. The website hosts a message board.

Further information about the training can be found in the summary of the workbook (Additional file 1).

### Treatment integrity

The trainers are instructed by two of the authors (WvB and AK). The training is structured along a treatment-manual and each session is being audio-taped and analyzed by one of the authors (WvB). The trainers fill in a form which states the main topics for each session in order to help them to stay focused on the manual.

### Control Condition

Participants in the control condition receive treatment as usual. Our training is additional and does not interfere with the ongoing treatment. In order to be able to compare the TAU and the TAU+ group we gather information on several characteristics of treatment as usual.

### Measurements

#### Sample characteristics

We gather information about demographic characteristics (age, marital status, education level) and parasuicidal behavior (self harming, past suicide attempts, risky behavior in traffic) and drug and alcohol abuse.

### Primary Outcome

#### Suicide ideation

With the Scale for Suicide Ideation [40] (SSI) we assess the presence and the level of suicide ideations. The SSI is a 19-item, clinician-administered semi structured interview which has demonstrated high reliability, with an internal-consistency coefficient (Cronbach's alpha) of 0.89, and a reported interrater reliability coefficient of 0.83.

### Secondary Outcomes

#### Depression

The Beck Depression Inventory BDI-II [41] is a self-administered 21 item self-report scale measuring supposed manifestations of depression. The BDI-II takes approximately 10 minutes to complete. Internal consistency for the BDI-II ranges from .73 to .92 with a mean of .86 [42]. The BDI-II has a split-half reliability co-efficient of .93.

#### Hopelessness

Hopelessness is to be measured with Beck's Hopelessness Scale [43] (BHS), a 20-item measure pertaining to the global experience of hopelessness, modified from a simple True/False format to a 5-point Likert-style rating system. It has a strong internal consistency (.81 to .90 in different studies).

#### Quality of Life

We administer the OQ-45 [44] (Outcome Questionnaire 45) to assess well-being. Quality of Life is an important measure in RCTs because an increase in patient's subjective well being motivates them to generalize what they learn during the treatment [45].

### Explanatory variables

#### Coping

The Coping Inventory for Stressful Situations [46] (CISS) is a 48-item self-report measure of coping. The measure is divided into three subscales, each containing 16 items: task-oriented coping, emotion-oriented coping, and avoidance-oriented coping. Respondents are asked to rate on a 5-point scale how each item is representative of their own way of coping with stress. The CISS has adequate psychometric properties. Across studies, the CISS has proved to be reliable. The internal consistency of the sub-scales is excellent (alpha > 0.85) [47].

#### Time Fluency

Our adapted version of MacLeod's Future Thinking Task [48] (FTT) is used to determine both positive and negative ideas about the past, present and the future. MacLeod's fluency task consists of three future time periods: the next week, the next year and the next five to ten years. Subjects are given 30 seconds to verbally provide examples for each time period: things they are looking forward to, and things they are not looking forward to. Our adapted version also inquires about current and past time periods, and assesses the emotional relevance of the experiences and their subjective significance for the future.

#### Time Perspective

Zimbardo's Time Perspective Inventory [49] (ZTPI) provides information about the time perspectives of the participants. The ZTPI consists of 56 items that are assessed on a 5-point Likert Scale, ranging from (1) very untrue to (5) very true. It has a high test-retest reliability ranging from .70 to .80 for the different factors.

#### Transcendental Future thinking

Another time related instrument is the additional scale of the ZTPI called the Transcendental Time Perspective Inventory (TFTPI), measuring what Boyd & Zimbardo [50] called transcendental future thinking: one's ideas about the afterlife as a motivating factor in one's present behaviour. The TFTPI consists of 10 statements. Participants rate these statement on a 5-point Likert scale (see ZTPI).

#### Social Problem Solving

The Social Problem-Solving Inventory-Revised [51] (SPSI-R) consists of 52 items that respondents rate on a 5-point scale. The SPSI-R has five scales: Positive Problem Orientation (PPO, 5 items), Negative Problem Orientation (NPO, 10 items), Rational Problem Solving (RPS, 20 items), Impulsivity/Carelessness Style (ICS, 10 items), and Avoidance Style (AS, 10 items). Alpha values for these five scales range from .76 to .92 and test-retest reliability ranges from .72 to .88.

#### Analyses

We are particularly interested in the effect (Cohen's *d*) on the main parameter suicide ideation. The effectiveness analyses will be conducted according to both intention-to-treat (ITT) and treatment completers principles. In the ITT analysis all randomized participants in the treatment group are included, irrespective of adherence, actual treatment received, or withdrawal from treatment or assessment. The completers analysis will focus on those participants who took part in 80% or more of the sessions and completed the post measurement.

Descriptive and mediating variables will be analyzed in order to reveal variables that need to be taken into account as covariates in the primary analyses of treatment effects. In order to find differences between the effects of our Future Oriented Group Training and treatment as usual we will perform analyses of repeated measures. We expect data loss due to drop out of participants. By using latent random effects variables for each participant multi level multivariable analysis permits estimation of changes in repeated measures, even when not all post assessment data are available due to missing data.

## Discussion

We have developed our Future Oriented Group Training based on the presumption that suicide ideation is characterized by diminished positive future thinking. Our intervention intends to stimulate realistic future perspectives. When suicidal individuals are able to envision a worthwhile future, their hopelessness and suicidal thinking and behavior are expected to decrease.

Extensive research the last twenty years has provided information about the different aspects and dynamics of suicidal thinking and behavior, but only a few interventions for suicidal patients have been evaluated in randomized clinical trials. The ones we know of (for instance Brown et al. [52]) have been developed for patients coming into care after a suicide attempt. Our training aims to help patients early on in the suicidal process, and we include both patients with suicidal ideation and after a suicide attempt in our study.

Future Oriented Group Training combines different elements that have proven to be effective in the treatment of suicidal thinking and behavior, like cognitive therapy and problem solving. Relatively new is the emphasis in the training on future thinking and goal oriented behavior. The intervention is designed to be easy to implement and is suitable for a broad range of comorbid psychiatric disorders.

Treatment programs like our training encompass several potentially effective elements. In the RCT we cannot distinguish which specific factor contributes to what extend to the overall treatment effect. This is also a characteristic of well established treatments, like Dialectical Behavioral Therapy [53]. We obtain an indication of changes in specific areas by gathering data on explanatory factors, like coping, problem solving, and future orientation, but we cannot tell which element of the training is responsible for these changes. Further research might be helpful to discriminate the efficacy of the separate elements.

Stimulating future thinking is a way of helping suicidal individuals to recreate a meaningful life, by working on purposeful goals and overcoming inefficient behavioral and cognitive patterns. The goal of our Future Oriented Group Training is to help our patients to make life livable and maybe even enjoyable again by realistically focusing on what the future might have to offer.

## Competing interests

The authors declare that they have no competing interests.

## Authors' contributions

WvB was responsible for the initial draft of this article, and the organization and implementation of the study. AK and AB contributed to the design and implementation, reviewed the workbook and manual, and revised earlier versions of the manuscript. All authors read and approved the final manuscript.

## Pre-publication history

The pre-publication history for this paper can be accessed here:



## Supplementary Material

Additional file 1 **Future oriented group training for suicidal patients: Description of the Intervention**. Provides some practical information about the intervention, and a case example. Click here for file
