# Supplementary material for: Future oriented group training for suicidal patients: a randomized clinical trial
Source: BMC Psychiatry. 2009 Oct 7;9:65. doi: 10.1186/1471-244X-9-65 (PMC2767345; doi:10.1186/1471-244X-9-65)
Supplement: Additional file 1 — Future oriented group training for suicidal patients: Description of the Intervention. Provides some practical information about the intervention, and a case example. [file 1471-244X-9-65-S1.DOC]

**Future Oriented Group Training for Suicidal Patients: Description of the Intervention**

(Additional file to **Future Oriented Group Training for Suicidal Patients: A Randomized Clinical Trial)**

W. van Beek^1^, A.J.F.M. Kerkhof, PhD ^,2^, A.T.F. Beekman, MD PhD^2^

^1^Symfora groep, Hilversum; EMGO Institute for Health and Care Research, Amsterdam, The Netherlands

^2^Vrije Universiteit, Amsterdam; EMGO Institute for Health and Care Research, Amsterdam, The Netherlands

Email addresses:

WvB: w.van.beek.hil@symfora.nl

AK: AJFM.Kerkhof@psy.vu.nl

AB: AartjanB@ggzingeest.nl

**Introduction**

Future Oriented Group Training is suitable for patients with suicidal thoughts, regardless of psychiatric comorbidity. Patients who are emotionally or cognitively dysregulated, like in a manic of psychotic state, are excluded from the project. The training is accessible for both inpatients and outpatients, and is suitable for general psychiatric settings. In our research project we randomize about two weeks before the actual start of the training. Half of the group of participants receive treatment as usual plus our add on training, the other half receives treatment as usual.
 The group consists of 4 to 8 patients, adults in the age range of 18-65 years. The training lasts 10 weeks, and has weekly 1,5 hours sessions. At the start of the training each of the participants receives a workbook, which contains texts with homework, and audio cd with additional exercises. The trainers use a treatment manual, which consist of the workbook with supplementary information on the background of the training and a checklist of topics to be discussed each session. All the trainers were introduced in the treatment by two of the authors (WvB and AK). The training was developed to be suitable for regular mental health care workers. Formal training in cognitive psychology or group therapy is not necessary.

*First session: Suicidality and Future Thinking*

The training begins with emphasizing that we will be talking about topics that underlie suicidality, but that we will keep on focusing on a how to work on a worthwhile future. We illustrate the main subjects of the training in terms of typical suicidal thinking or behavior, and subsequently focus on desired cognitions and efficient action. Some participants need time to emphasize that their suicidal ideas are based upon their personal experiences and how ‘true’ they are. We acknowledge and validate the reasons participants have for their current ways of thinking and behaving, but we also stress the need for change. This is a good opportunity to discuss efficient and effective thinking and behavior.
 During the first session we explain relationships between suicidality, negative expectation and diminished future thinking in suicidal people. Suicidality is related to the prediction that the future will be negative and particularly that there are hardly any positive experiences to look forward to. We explain how people construct their own expectations, and heavily base those expectations upon prior generalized experiences.
 We introduce the concept of coaching oneself, and organizing an external personal coach or buddy. Participants are strongly advised to seek someone who can be their support during the training, like a friend or a partner. These personal coaches receive a workbook. They can also attend a session to experience what this training is about, actively or more passively. Although some participants express their worries about not being able to find someone, most of them actually do. This is a moment for us to discuss the tendency of suicidal individuals to isolate themselves. One of the positive reactions we receive when we evaluate the training is that this is the first time for some participants and coaches to talk about their suicidality while they are not in a crisis situation. They learn not to be too frightened by the subject and discuss how to be able to talk about it with someone they care about. We are currently gathering a pool of volunteers who can be asked to serve as a coach when the participants have no one they can or dare to ask.

*Session 2 and 3: Change*

At the start of every session we discuss the homework and participants comment to the cd. Listening to the audio cd takes about 20 minutes per week, doing the homework assignments about the same. Both the audio and the paper assignments focus on aspects of the training previously discussed. The first exercise in the workbook deals with imagining change, and we ask the participants what they would want to change in their lives, without thinking about how to manage that. A couple of exercises in the workbook focus on practicing envisioning different outcome scenarios. We ask the patients to imaginary create different scenarios, and experience their tendency to believe that only the negative ones are true. How they defend this hopeless stance without actually wondering if they should. We discuss automated thinking and behavior, cognitive reflexes, and one’s tendency to resist against change.
 We take a sidestep to stimulate positive and constructive thinking and behavior. The participants fill in a questionnaire asking for personal competencies and the workbook lists some exercises from positive psychology, like doing small favors to feel better about oneself. All of these texts and homework assignments are meant to help participants to start feeling in control of their own lives more than they did.

*Session 4 and 5: Cognitive therapy*

In this phase participants appear to becoming aware of the role of their thoughts, believes and expectations in their hopelessness. Patients are taught to recognize that their feelings and behavior are at least influenced by their cognitions, and that it might be useful to question the efficiency of these thoughts. During session 4 and 5 we teach the patients about inadequate cognitive styles and they fill in a questionnaire that helps them to differentiate their cognitive schemas. Once again we focus on those ideas that obstruct future goals, maladaptive cognitive patterns like ‘I will not be able to finish this training’, or ‘This treatment is not sufficient because I am a hopeless case’. Which thoughts and convictions are automatically triggered when you start raising hope? What comes up in your mind when you set goals and strive to change something? And how can we help you to protect yourself against these destructive cognitive tendencies? In our training we teach participants to recognize their hopelessness invoking thoughts in order to safeguard themselves against these ideas when they start making plans again.

*Session 6 and 7: Goal Orientation*

At this point in the training most participants are becoming aware of their avoidance, their isolation, their perfectionism or fear of failure, and some of their inefficient cognitions. Then we talk about change, about making plans, and taking steps to achieve one’s goals. In the workbook we differentiate steps and means, as is being done in problem solving therapy. What do you need, who can support you, and which tendencies do your need to be aware of when you start to make plans again? We discuss goal formulation and achievable goals, high expectations and avoidance as a way of guarding against more unbearable negative feelings like failure. We talk about successful people, like in sports, what is necessary to achieve one’s goals? And what is the role of rewards and support from significant others? Some of these ideas are based upon Problem Solving Therapy, but we do not focus on problems, but on desired outcomes.

*Session 8 and 9: Taking Steps*

In this phase we combine the previously discussed elements in a structured format. The workbook provides a document in which (a) personal meaningful goals, (b) motivation, (c) cognitive and behavioral pitfalls, (d) steps/sub goals, and (e) means can be formulated. Each participant focuses on his own goal and determines how to start an experiment with alternative behavior, taking into account what one has learned during the training. The group members discuss their own plan and reflect on each other’s ideas. Once again the focus is on how to accomplish desired outcome. One of the benefits of the group character is that participants stimulate one another and can give practical information that can be helpful. In one of our groups a patient wanted to pick up a study and a group member supported her in doing so, by providing necessary information on a specific course.
The format participants can use when they start working on a personal goal consists of 13 steps and 5 additional points of attention. This form summarizes what we discussed in the training, and can be used during the training and afterwards. Participants can download it from our website when they need additional copies.

The content of the training are summarized in these questions, part of the written plan participants fill in:

*“Tell me which goal you want to reach. Can you explain me why this is important and how it relates to making your life more meaningful again. Which thoughts will show up as a threat to reaching your goal. Which thoughts motivate you and help you. How can your personal coach assist you in this. Which sub goals are necessary. What do you need to do (steps), and which tools do you need. Describe your time plan. What will you need to do when something demotivates you, when you get frustrated (e.g. support, relaxation). Which competencies can you use in order to reach your goal. What do you need from others. How can you reward yourself during the process of working on your goal. What is effective and what are ways of being more effective when it comes to this goal. Describe a detailed imaginary situation in which you reached your goals; what does it look like. And keep in mind: which alternative goals and steps am I leaving out. What in your plan so far would I not recommend to my friend. Are the steps realistic (80% sure it will work). Is it encouraging, helpful, fun, and challenging enough”*

*Session 10: Consolidation and evaluation*

During the last session we talk about ways to preserve what the patients learned in the training. We advise participants to read the workbook regularly, and to use their personal coach to encourage them to do so. We talk about what each participants expects to happen, which cognitions and typical behavior will keep them from doing so. Part of the conclusion is the structured evaluation of the treatment.

*Case: Michelle the accountant*

Patient is a 34 years old female with recurrent suicidal periods. She remembers that she had her first suicidal thoughts when she was 11, after her grandfather died. Her first few suicidal episodes occurred after an emotional experience, but later on these ideas came back without a recognizable reason. She enters treatment because she feels depressed and has problems maintaining close relationships. She works as an accountant and has currently no relationship. Patient hesitates about participating in the training, she does not want to talk about her suicidality, because she wants to feel better en find a cure for her depression. She understands our explanation about the reoccurrence of her suicidal thoughts and the need for specifically treating this. She feels she is different from the typical suicidal patients though.

Michelle finds it a relief that we are going to talk about future goals, but she objects against the statement of the trainer that her suicidality is related to her way of thinking. She has very good reasons why she is depressed and suicidal, and feels the need to tell us these reasons. The trainer validates her story, and then generalizes the content, particularly about her perfectionism. Michelle gets support and understanding from other participants. She acknowledges that there are a lot of automated thoughts in her head, and that she feels a strong inner drive to succeed, but that she lost the self-confidence to keep up with her own demands. When it comes to finding a coach to support her during the training, she is convinced that she will not find anyone. Her best friend Mary would be too busy, and Michelle is be reluctant even to ask, because this friend helped her out before when she was ‘really suicidal’. After our explanation that it would be helpful for both of them to be able to talk about suicidal thinking before it comes to a crisis Michelle agreed that she might ask Mary. The next session she did not. During this session we talk about social isolation among suicidal patients, the loss of social connections, and the inability to ask for help in a meaningful way. The next session Michelle tells that she did ask her friend and that Mary agreed to help. They even planned meetings to talk about the training.
 After that we discuss helpful and unhelpful thoughts in the training. Michelle fills in a form and learns that the need to do better than others is an important cognition. When we talk about she tells us that she is never successful enough, which might have to do with parental messages. Like almost everything we discuss we relate this thought to future meaningful goals and subsequent hopelessness. Telling herself that she needs to do better might be encouraging when she feels good, but it is destructive when she feels depressed. We emphasize the need to think in terms of being successful. That Michelle should ask herself when these cognitions are helpful, and when not.
 We then talk about coaching oneself. Michelle has been a successful tennis player and she knows what she needs to reach goals: both encouragement and validation. She needs to be told that she is doing well, but that she could do better. She realizes that she finds it hard to ask others for support. During these sessions she recognizes cognitive patterns that consolidate her hopelessness. The following meetings Michelle formulates goals that would make her life meaningful again. Her goals are different from what others formulate. Michelle’s aim is to go looking for a partner again, whereas others in the group formulate goals like ‘resolving the conflict with my mother’, or ‘get rid of my financial problems’. Michelle then starts working on this one goal (she had more, like most other participants). Recognizing that she not only held high standards for herself, but also for potential partners. And predicting that no would actually want to date a 34 years old woman with psychological problems. There is a huge gap between what she considers to be successful, and what she actually accomplishes.
 The last few sessions she integrates what she has learned about her thinking style, her vulnerabilities, and about successful behavior (and how she acts to not be successful). The group encourages her to take the first steps and much to her own surprise she can encourage herself, with the help of her coach, to start dating again – with different expectations. During the evaluation session Michelle states that she feels she is more in control of her own life than before. She feels less hopeless, although there is a lot to be changed. She can describe what she has (not) to do, and what she should (not) to say to herself, to start feeling suicidal again. The trainers emphasize that she possibly might start feeling suicidal again, but that she has gathered tools to protect herself from passively following these patterns. And that successfully working on the goals that make life worthwhile again guards her against the hopelessness that comes along with suicidal thinking.

*Discussion*

Suicidal thinking and behavior encompass a wide array of influences and causes, and are obviously closely related to existential themes. This complexity forces us to limit the scope of any intervention, and in our training we addressed some of the most acknowledged aspects in suicidality. Particularly underlying hopelessness, and more specific: lack of positive future thinking. Our training hopes to help patients to recreate this worth once again, by specifically focusing on what in one’s personal future would make life worthwhile again. And helping participants to be able to be working on those meaningful goals again, taking their cognitive and behavioral pitfalls into account.
